# A computational approach to simulating a three-sphere swimmer in a viscoelastic fluid modelled via the Giesekus constitutive law

**DOI:** 10.1098/rsta.2024.0268

**Published:** 2025-09-11

**Authors:** Cara Victoria Neal, Rachel Naomi Bearon

**Affiliations:** ^1^ Department of Mathematics, University College London, London, UK; ^2^ Department of Mathematical Sciences, University of Liverpool, Liverpool, UK; ^3^ Department of Mathematics, King's College London, London, UK

**Keywords:** microswimmers, viscoelasticity, finite element methods, shear-thinning, non-Newtonian

## Abstract

Microswimmer locomotion in non-Newtonian fluids is crucial for biological processes, including infection, fertilization and biofilm formation. The behaviour of microswimmers in these media is an area with many conflicting results, with swimmers displaying varying responses depending on their morphology, actuation and the complex properties of the surrounding fluid. Using a hybrid computational approach, we numerically investigate the effect of shear-thinning rheology and viscoelasticity on a simple conceptual microswimmer consisting of three linked spheres. Our approach utilizes known Newtonian solution methods (Cortez 2001 The method of regularized Stokeslets (MRS). *SIAM J. Sci. Comput*. **23**, 1204–1225. (doi:10.1137/s106482750038146x)) to approximate the rapidly varying flow surrounding the swimmer, with a non-Newtonian correction obtained via the finite element method (FEM). The problem is formulated such that the solution can be calculated over a coarse mesh of the fluid domain, meaning accurate results can be obtained for low computational costs. Our results demonstrate enhancements in swimming speed and efficiency of up to 7 and 16%, respectively, for locomotion in non-Newtonian versus Newtonian fluids. We discuss how this computational approach could further be used to model bio-inspired swimmers and explain the transitions between the apparently contradictory results in the literature.

This article is part of the theme issue ‘Biological fluid dynamics: emerging directions’.

## 1. Introduction

In their natural biological environment, microswimmers often swim in complex fluids with non-Newtonian characteristics. Examples include mammalian spermatozoa swimming in mucosal media in the cervix or along the fallopian tubes [1–3], the bacterium *Helicobacter pylori* moving through the mucus layer covering the stomach and causing ulcers [4,5], the Lyme disease-causing spirochete *Borelia burdorferi* penetrating the connective tissues in our skin [6,7] and bacteria producing extracellular polymeric substances and forming biofilms [8,9]. On the scale of a single cell, these mucosal fluids are not continuous; rather, they are a network of long polymer chains suspended within solution [10]. The presence of polymers endows the mucus with complex properties that can affect a swimmer’s progress through it.

Two non-Newtonian fluid properties are likely to influence microswimmer motility: shear-thinning rheology and viscoelasticity [11,12]. A viscoelastic fluid exhibits elastic characteristics when undergoing deformations, whereas the viscosity of a shear-thinning fluid decreases with increased fluid shear rates. There has recently been a lot of interest in how non-Newtonian fluids influence microswimmer propulsion compared to swimming in a Newtonian fluid. Various theoretical and experimental studies reveal scenarios where the swimming speed of micro-organisms increases [13–23], decreases [24–30] or remains unchanged [31–34] in a non-Newtonian fluid, with the exact response depending largely on the type of swimmer, swimming gait and non-Newtonian effects being considered. Many of these results appear to be apparently conflicting and most consider the effects of only shear-thinning rheology or viscoelasticity, despite the fact that most biological fluids exhibit both properties. Furthermore, many simulations focus on small-amplitude asymptotics [34,35] due to the complexity and computational costs of simulations of large-amplitude motion in fluids described by nonlinear equations. The exceptions to this [13,17,36] provide important novel physical insight but tend to be very computationally expensive and difficult to extrapolate to other swimmer morphologies or fluid properties. It would therefore be useful to have a modelling tool that is extensible, computationally efficient and able to explore both the effect of shear-thinning rheology and viscoelasticity on microswimmer propulsion.

In this paper, we introduce the hybrid approach for nonlinear swimming (HANS) and demonstrate how HANS can be used to efficiently and accurately solve flow problems involving a microswimmer in a two-dimensional shear-thinning viscoelastic fluid. The HANS method was introduced in previous work to simulate sperm in purely shear-thinning fluids [37]. The method consists of constructing a Newtonian approximation to the flow field using the method of regularized Stokeslets (MRS) [38], a convenient and popular method for solving biological flow problems involving microswimmers. We then ‘correct’ for the non-Newtonian effects of the fluid through solving a modified flow problem using the finite element method (FEM). In this way, the overall outcome is an accurate solution to the full nonlinear flow problem. This application of HANS constitutes the first use of the MRS for problems involving viscoelastic fluid flow. Since the regularized Stokeslet accounts for the rapidly varying nature of the flow close to the swimmer, the correction term is slowly varying across the domain. Consequently, the FEM solver can be formulated on a coarse mesh rather than a body-fitted mesh as required by previous numerical solvers for swimmers in non-Newtonian fluids [13], for increased computational efficiency.

We model the conceptual Najafi–Golestanian swimmer [39], which consists of three linked spheres that move relative to each other with non-reciprocal motion to induce net displacement. The simplicity of this swimmer allows us to clearly define the model formulation and implementation of the HANS method, with modelling more complicated swimmers presenting a natural extension. After outlining the solution method and verifying the numerical solver, we analyse how the trajectory of the swimmer is influenced by the fluid and investigate how both fluid elasticity and shear-thinning effects influence both the swimming speed and efficiency. Finally, we examine the flow and stress fields surrounding the swimmer to undercover the physical mechanisms behind the changes in propulsion observed.

## 2. Fluid mechanics at low Reynolds number

Microscopic swimmers experience a low-Reynolds-number regime where viscous forces dominate inertial forces. For steady incompressible low-Reynolds-number flow, the momentum and continuity equations are written as


(2.1) $$\begin{array}{lcr} & \nabla \cdot \sigma +F=0,\;\nabla \cdot u=0, & \end{array}$$

where u=u(x,t) is velocity, x is position, t is time, F is the body force acting on the fluid and σ=σ(x,t) is the stress tensor, the form of which depends on the type of fluid being modelled.

In the case of a Newtonian fluid, stress is proportional to strain rate, and the form of the stress tensor is given by


(2.2) $$\begin{array}{lcr} & \sigma =-pI+2\eta D(u). & \end{array}$$

Here, p=p(x,t) is fluid pressure, I is the identity tensor and η is the dynamic viscosity of the fluid. The strain-rate tensor, D(u), is defined as


(2.3) $$\begin{array}{lcr} & D(u)=\frac{1}{2}\left(\nabla u+(\nabla u{)}^{T}\right). & \end{array}$$

Substituting the equation for the stress tensor into the system (2.1) yields the equations for a Newtonian fluid at low Reynolds number, known as the Stokes flow equations:


(2.4) $$\begin{array}{lcr} & \eta \nabla^{2}u-\nabla p+F=0,\;\nabla \cdot u=0. & \end{array}$$

The Stokes flow equations have several properties that have important consequences when modelling microswimmers in Newtonian fluids. The first of these is linearity, meaning that solutions can be constructed via any finite sum of solutions to the system (2.4). An example of this in application is Hancock’s slender body theory [40], in which the flagellum of a sperm cell is represented via a line integral of point forces, called Stokeslets.

The Stokes flow equation (2.4) also has no explicit dependence on time, meaning that the flow is instantaneously determined by the boundary conditions of a given problem. As a consequence of this, Purcell’s scallop theorem states that swimmers must employ a time-irreversible swimming stroke to generate net displacement in Stokes flow [41].

In this paper, we will simulate a simple swimmer in a non-Newtonian fluid environment exhibiting both shear-thinning and viscoelastic properties. In this case, the Stokes flow equations no longer apply, with the new equations taking a nonlinear form in which the scallop theorem no longer holds true [28,42]. However, using the HANS method, we demonstrate how we can utilize Newtonian solution techniques to solve non-Newtonian swimming problems, specifically using the MRS [38]. In §2a, we introduce the equations of flow for fluids exhibiting both shear-thinning and viscoelastic effects. We then proceed to introduce the Stokeslet solution and the MRS in §2b. Following this, we outline the swimmer model and solution technique HANS in §3 and verify the numerical implementation through comparison with a semi-analytic solution for a simplified problem.

### (a) Governing equations for shear-thinning viscoelastic fluids

For the non-Newtonian fluids we will consider, the form of the stress tensor is given by


(2.5) $$\begin{array}{lcr} & \sigma =-pI+\tau , & \end{array}$$

where τ=τ(x,t) is the polymeric stress tensor. The equations of flow are therefore given by


(2.6) $$\begin{array}{lcr} & -\nabla p+\nabla \cdot \tau +F=0,\;\nabla \cdot u=0. & \end{array}$$

To close the model, a transport equation for the polymeric stress is required. Here, we adopt the nonlinear Giesekus model [43], which, in addition to displaying shear-thinning material properties, provides two important features, namely, saturation of polymer elongation and a non-negative entropy production during the time evolution of the polymers (see [44–46] for details). The constitutive equation for this model can be written as


(2.7) $$\tau +\frac{\alpha \lambda }{\eta }(\tau \cdot \tau )+\lambda \overset{\;▽}{\tau }=2\eta D(u),$$

where $\overset{\;▽}{\tau }$ denotes the upper-convected derivative, defined for a tensor τ as


(2.8) $$\overset{\;▽}{\tau }=\frac{D\tau }{Dt}-\left(\tau \cdot \nabla u+{\left(\nabla u\right)}^{T}\cdot \tau \right)\;\text{ where }\;\frac{D\tau }{Dt}=\frac{\partial \tau }{\partial t}+\left(u\cdot \nabla \right)\tau .$$

Here, Dτ/Dt is the material derivative of the stress field, and the model parameters λ and η represent the fluid relaxation time and viscosity, respectively. The dimensionless mobility factor 0≤α≤1 characterizes the fluid’s shear-thinning behaviour and represents the anisotropic hydrodynamic drag exerted on the polymer molecules by the surrounding solute molecules [47]. Here, α=1 represents maximum anisotropy, while α=0 corresponds to the isotropic relaxation of the Oldroyd-B model, which does not display shear-thinning behaviour. Physically, α quantifies the capability of polymer chains to be stretched along with the flow. From thermodynamics considerations, the mobility factor should be kept in the 0–0.5 range [47,48].

### (b) MRS in two dimensions

The fundamental solution to the Stokes flow equation (2.4) driven by a point force will form an important part of our solution method. Consider a single point force of magnitude f acting at the point $y\in {\mathbb{R}}^{2}$ within a Stokesian fluid. The total body force acting at a point $x\in {\mathbb{R}}^{2}$ is given by


(2.9) $$\begin{array}{lcr} & F(x)=f\delta (x-y), & \end{array}$$

where δ is the two-dimensional Dirac delta distribution, which is infinite at x=y, and where


(2.10) $$\begin{array}{lcr} & \begin{array}{r} \iint_{D}\delta (x-y)dx=\left\{ \begin{array}{cc} 1 & \text{ if }y\in D\subset {\mathbb{R}}^{2},\\ 0 & \text{ if }y\notin D. \end{array}\right. \end{array} & \end{array}$$

The solutions to the Stokes flow equations with F defined via equation (2.9) are given by


(2.11) $$\begin{array}{lcr} & u_{i}(x)=S_{ij}(x,y)f_{j}(y),\;p(x)=P_{j}(x,y)f_{j}(y), & \end{array}$$

which are known as the Stokeslet solutions. Here, repeated indices are summed over and


(2.12) $$\begin{array}{lcr} & S_{ij}(x,y)=\frac{1}{4\pi \eta }\left(-\delta_{ij}\ln(r)+\frac{r_{i}r_{j}}{r^{2}}\right),\;P_{j}(x,y)=\frac{1}{2\pi }\left(\frac{r_{j}}{r^{2}}\right), & \end{array}$$

for a fluid of viscosity η, where $r_{i}=x_{i}-y_{i}$ and r=|x−y|. The rank-2 tensor S is known as the *Stokeslet* (or the Oseen tensor), a term first coined by Hancock [40].

In addition to the velocity and pressure solutions discussed above, the stress field associated with the flow can be defined in a similar way via


(2.13) $$\begin{array}{lcr} & \sigma_{ik}(x)=T_{ijk}(x,y)f_{j},\;T_{ijk}(x,y)=-\delta_{ik}P_{j}(x,y)+\eta \left(\frac{\partial S_{ij}(x,y)}{\partial x_{k}}+\frac{\partial S_{kj}(x,y)}{\partial x_{i}}\right), & \end{array}$$

where the rank-3 stress tensor T is sometimes termed the *stresslet*.

These solutions are singular at r=0, which presents a challenge when considering numerical approaches to solving problems. A popular approach that alleviates this complication is the MRS, proposed by Cortez [38]. The regularized Stokeslet is the solution to the Stokes flow equation (2.4), with a spatially smoothed body force defined via


(2.14) $$\begin{array}{lcr} & F(x)=f\phi_{\epsilon }(x-y)\;\text{ where }\;\int_{{\mathbb{R}}^{2}}\phi_{\epsilon }(x)\text{d}x=1. & \end{array}$$

The function $\phi_{\epsilon }(x)$ is known as the blob function and tends to the Dirac delta distribution as ϵ→0. A popular choice in two dimensions, which we use from now on, is given by


(2.15) $$\begin{array}{lcr} & \phi_{\epsilon }(x)=\frac{3\epsilon^{3}}{2\pi (r^{2}+\epsilon^{2}{)}^{5/2}}, & \end{array}$$

where the regularization parameter 0<ϵ≪1 controls the radius of the smoothed blob over which the force is applied. For this $\phi_{\epsilon }$, an analogue of the Stokeslet solution (equation (2.12)), the regularized Stokeslet, can be derived. The regularized Stokeslet velocity and pressure tensors are, respectively, given by


(2.16a)Sijϵ(x,y)=14πη(−δijln⁡(r2+ϵ2+ϵ)+δijϵ(r2+ϵ2+2ϵ)(r2+ϵ2+ϵ)r2+ϵ2+rirj(r2+ϵ2+2ϵ)(r2+ϵ2+ϵ)2r2+ϵ2),(2.16b)Pjϵ(x,y)=12π(rj(r2+2ϵ2+ϵr2+ϵ2)(r2+ϵ2+ϵ)(r2+ϵ2)3/2),

where $r_{i}=x_{i}-y_{i}$ and r=|x−y|. Similarly, the regularized stresslet, $T_{ijk}^{\epsilon }(x,y)$, can be calculated using the same relation as in equation (2.13). These regularized Stokeslet solutions will form part of our solution method for the nonlinear swimming problem.

## 3. Model formulation

**Figure 1.**
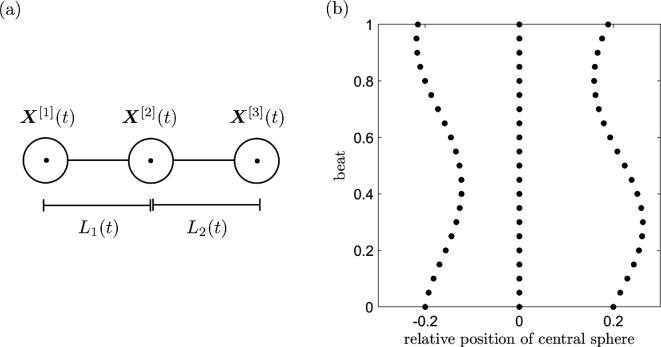
(a) Schematic of the three-sphere swimmer represented by regularized Stokeslets at $X^{\left[1\right]}(t)$, $X^{\left[2\right]}(t)$ and $X^{\left[3\right]}(t)$, connected by springs of lengths $L_{1}(t)=|X^{\left[2\right]}(t)-X^{\left[1\right]}(t)|$ and $L_{2}(t)=|X^{\left[3\right]}(t)-X^{\left[2\right]}(t)|$. (b) Position of the spheres relative to the central sphere over a beat.


We consider in this paper a simple model of a viscous swimmer, as proposed by Najafi & Golestanian [39], comprising two outer ‘spheres’ that move relative to a central ‘sphere’ (note that, since we are working in two dimensions, these ‘spheres’ are actually circles). We enforce a non-reciprocal swimming gait that avoids violating Purcell’s scallop theorem [41], allowing net motion in both Newtonian and non-Newtonian fluids and enabling a comparison of locomotion in these environments. We represent the swimmer through three collinear regularized Stokeslets with time-varying locations $X^{\left[1\right]}(t)$, $X^{\left[2\right]}(t)$ and $X^{\left[3\right]}(t)$ (figure 1*a*). The spheres are connected via two Hookean springs of lengths $L_{1}(t)=|X^{\left[2\right]}(t)-X^{\left[1\right]}(t)|$ and $L_{2}(t)=|X^{\left[3\right]}(t)-X^{\left[2\right]}(t)|$ and resting length ℓ. In this way, a restoring spring force per unit length is experienced by the spheres, given (for the two outer spheres) by

(3.1) $$f_{H}^{\;[1]}(t)=K\left(L_{1}(t)-\ell \right)\bar{d_{1}}(t),\;f_{H}^{\;[3]}(t)=-K\left(L_{2}(t)-\ell \right)\bar{d_{2}}(t),$$

where K is a constant related to the stiffness of the springs, and d1(t) and d2(t) are directional vectors given by d1(t)=[X[2](t)−X[1](t)]/L1(t) and d2(t)=[X[3](t)−X[2](t)]/L2(t).

Motion of the two outer spheres is induced by applying the forces per unit length:


(3.2) $$f_{A}^{\;[1]}(t)=KA\sin(2\pi \omega t)\bar{d_{1}}(t),\;f_{A}^{\;[3]}(t)=-KA\sin(2\pi \omega t-\chi )\bar{d_{2}}(t),$$

where A and ω are the amplitude and frequency of the applied beat force, respectively, and symmetry is broken by enforcing the phase difference χ. The total force per unit length acting on the outer spheres is given by $f^{\;[1]}(t)=f_{A}^{\;[1]}(t)+f_{H}^{\;[1]}(t)$ and $f^{\;[3]}(t)=f_{A}^{\;[3]}(t)+f_{H}^{\;[3]}(t)$. By Newton’s third law $f^{\;[2]}(t)=-\left(f^{\;[1]}(t)+f^{\;[3]}(t)\right)$, and the swimmer is force-free. The position of the two outer spheres relative to the central sphere over a beat cycle is plotted in figure 1b.

The swimmer is situated within a two-dimensional rectangular domain $\Omega \subset {\mathbb{R}}^{2}$ with boundary ∂Ω, occupied by a fluid whose rheological behaviour is modelled via the Giesekus constitutive law. We prescribe the no-slip condition u(x,t)=0 on ∂Ω and non-dimensionalize via a characteristic length scale L, time scale $\omega^{-1}$, force scale KL, velocity scale Lω and pressure/stress scale ηω. The dimensionless boundary value problem (BVP) is therefore given via equations (2.6) and (2.7) with F defined in equation (2.14), by (dropping the x and t dependence for brevity)


(3.3a)−∇p+∇⋅τ+κ∑m=13f[m]ϕϵ(x−X[m])=0,x∈Ω,(3.3b)∇⋅u=0,x∈Ω,(3.3c)τ+αWi(τ⋅τ)+Wiτ∇=2D(u),x∈Ω,(3.3d)u=0,x∈∂Ω,

where κ=K/ηω and the dimensionless strain-rate tensor D(u) and regularized Stokeslet cutoff function $\phi_{\epsilon }(x)$ are defined as previously in equations (2.3) and (2.15), respectively. The dimensionless forces per unit length acting on the spheres are given by


(3.4a)f[1]=(L1−ℓ+Asin⁡(2πt))d1(t)¯,(3.4b)f[2]=−f[1]−f[3],(3.4c)f[3]=−(L1−ℓ+Asin⁡(2πt−χ))d2(t)¯,

where $L_{1}$, $L_{2}$, ℓ, A and ϵ represent the dimensionless versions of the original parameters that have been scaled by L. The Weissenberg number, Wi=λω, and shear-thinning index, α, determine the rheological properties of the fluid.

### (a) Numerical solution method

The solution of the BVP given in the system (3.3) is difficult to obtain for two key reasons: (i) the problem is nonlinear in the stress τ in the Giesekus constitutive law, and (ii) due to the presence of the regularized forces in the domain, the solutions p, u and τ vary rapidly close to the swimmer. The nonlinearity of the problem necessitates its solution via a numerical approach; however, the rapidly varying nature of the flow means that such attempts, for example, using the FEM to solve the system (3.3) directly, are likely to be very computationally expensive. To combat the issue of rapidly varying solutions, we introduce HANS. HANS was previously developed to solve problems involving swimming in shear-thinning fluids [37], and here we outline how it can be formulated and implemented to solve problems involving swimming in fluid modelled via the Giesekus constitutive law.

HANS is applied to the system (3.3) through first decomposing the solutions for pressure, velocity and stress into two components each via


(3.5) $$p(x,t)=P(x,t)+p_{N}(x,t),\;u(x,t)=U(x,t)+u_{N}(x,t),\;\tau (x,t)=T(x,t)+\tau_{N}(x,t).$$

The solutions $p_{N}(x,t)$, $u_{N}(x,t)$ and $\tau_{N}(x,t)$ form a Newtonian approximation to the flow surrounding the swimmer via the MRS. By construction, these solutions are rapidly varying close to the swimmer. In this way, they account for the majority of the rapidly varying nature of the overall fluid pressure p(x,t), velocity u(x,t) and stress τ(x,t) solutions over the domain. This means that the terms P(x,t), U(x,t) and T(x,t), which correct for the non-Newtonian effects of the fluid, will be slowly varying across the domain. This will be demonstrated following outlining the solution method. We employ the FEM to solve for these unknowns over a coarse mesh of the domain Ω. In this way, the HANS method greatly reduces computational costs by enabling accurate flow solutions to be obtained over a mesh consisting of a low number of elements ( less than 200), compared to what would be required to directly solve the system (3.3) numerically.

The Newtonian approximations solve the Stokes flow problem for a fluid of viscosity η in the infinite domain. Following §2b, these solutions are given by


(3.6a)pN(x,t)=∑m=13Pjϵ(x,X[m](t))fj[m](t),(3.6b)uNi(x,t)=∑m=13Sijϵ(x,X[m](t))fj[m](t),(3.6c)τNik(x,t)=∑m=13Tijkϵ(x,X[m](t))fj[m](t),

where $P^{\varepsilon }$, $S^{\epsilon }$ and $T^{\epsilon }$ represent the non-dimensionalized form of the regularized solution kernels. Given we know the forces ${\left\{f^{\;[m]}(t)\right\}}_{m=1}^{3}$ at each t based on the configuration of the swimmer, these solutions are known and do not need to be solved for.

To form a system to solve for the unknown non-Newtonian corrections, we substitute the expressions in equation (3.5) into the system (3.3). Exploiting the linearity of the strain-rate tensor, divergence operator and gradient operator, this yields


(3.7a)−∇P+∇⋅T+(∇⋅τN−∇2uN)=0,x∈Ω,(3.7b)∇⋅U=0,x∈Ω,(3.7c)T+αWi((T+τN)⋅(T+τN))+Wi∂(T+τN)∂t+Wi((U+uN)⋅∇)(T+τN)(3.7d)−Wi((T+τN)⋅(∇(U+uN))+(∇(U+uN))T⋅(T+τN))=2D(U),x∈Ω,(3.7e)U=−uN,x∈∂Ω,

where we have used that $u_{N}$ and $p_{N}$ satisfy the Stokes flow equation (2.4) to simplify equations (3.7a) and (3.7b), and we have simplified equation (3.7d) since the Newtonian solution satisfies $\tau_{N}=2D(u_{N})$. Given known solutions $p_{N}$, $u_{N}$ and $\tau_{N}$ at a given time t, we solve the system (3.7) using the FEM for P, U and T. Since the problem is nonlinear, we apply an iterative approach.

To derive the finite element discrete weak formulation, we consider a partition of the domain Ω into arbitrary quadrangles. This discretized domain is denoted $\Omega_{h}$ for mesh size h. We consider a velocity–pressure–stress formulation with our choice of discrete functional spaces based on previous analysis of both Newtonian and viscoelastic flows [48,49]. These spaces are defined as


(3.8a)Vhd={vh∈H1(Ωh;R2):vh=−uN on ∂Ωh},(3.8b)Vh0={vh∈H1(Ωh;R2):vh=0 on ∂Ωh},(3.8c)Qh={qh∈L2(Ωh;R2):∫Ωhqhdx=0},(3.8d)Xh={θh=(θij)1≤i,j≤2∈L2(Ωh;R2)},

where $H^{1}(\Omega_{h};{\mathbb{R}}^{2})$ is the Sobolev space of order 1 and $L^{2}\left(\Omega_{h};{\mathbb{R}}^{2}\right)$ is the space of square-integrable functions on $\Omega_{h}\subset {\mathbb{R}}^{2}$. Multiplying equation (3.7) by the test functions $v_{h}\in V_{h}^{\;0}$, $q_{h}\in Q_{h}$ and $\theta_{h}\in X_{h}$, respectively, and integrating by parts yields the discrete weak formulation:


$$\begin{array}{r} \left\{ \begin{array}{ll} (U_{h},P_{h},T_{h})\in V_{h}^{d}\times Q_{h}\times X_{h}\\ -a(P_{h},v_{h})+b(T_{h},v_{h})+d(v_{h}) & =0\;\forall v_{h}\in V_{h}^{\;0},\\ a(q_{h},U_{h}) & =0\;\forall q_{h}\in Q_{h},\\ c_{0}(T_{h},\theta_{h})+\alpha \text{Wi}\;c_{1}(T_{h};T_{h},\theta_{h})+\text{Wi}\;c_{2}\left(\frac{\partial (T_{h}+\tau_{N})}{\partial t},\theta_{h}\right)+\text{Wi}\;h_{0}(U_{h};T_{h},\theta_{h})\\ -\text{Wi}\;h_{1}\left(U_{h};T_{h},\theta_{h}\right)-2b\left(\theta_{h},U_{h}\right) & =0\;\forall \theta_{h}\in X_{h}, \end{array}\right. \end{array}$$

where


(3.9a)a(qh,vh)=∫Ωhqh∇⋅vhdx,(3.9b)b(θh,vh)=∫Ωhθh:D(vh)dx,(3.9c)d(vh)=∫Ωh(τN:D(vh)−∇uN:∇vh)dx(3.9d)c0(Th,θh)=∫ΩhTh:θhdx,(3.9e)c1(Th,Th,θh)=∫Ωh((Th+τN)⋅(Th+τN)):θhdx,(3.9f)c2(∂(Th+τN)∂t,θh)=∫Ωh∂(Th+τN)∂t:θhdx,(3.9g)h0(Uh;Th,θh)=∫Ωh((Uh+uN)⋅∇)(Th+τN):θhdx,h1(Uh,Th;θh)=∫Ωh((Th+τN)⋅(∇(Uh+uN))(3.9h)+(∇(Uh+uN))T⋅(Th+τN)):θhdx,

We approximate the time derivative through a backwards Euler scheme and rearrange the expressions in the weak formulation for a known r.h.s to give


(3.10a)−a(Phs,vh)+b(Ths,vh)=−d(vh),(3.10b)a(qh,Uhs)=0,Δtc0(Ths,θh)+αWiΔtc1(Ths;Ths,θh)+Wic2(Ths+τNs,θh)+WiΔth0(Uhs;Ths,θh)(3.10c)−WiΔth1(Uhs;Ths,θh)−2Δtb(θh,Uhs)=Wic2((Ths−1+τNs−1),θh),

where the superscript s represents the timestep index, and the initial condition is chosen to be the solution to the steady problem. To solve for the non-Newtonian solutions at each s, we apply velocity, pressure and stress expansions of the form $\;U_{i;h}^{s}=\sum_{n=1}^{N_{u}}U_{i;n}^{s}\phi_{n}(x)$ for i=1,2, $P_{h}^{s}=\sum_{n=1}^{N_{p}}P_{n}^{s}\psi_{n}(x)$ and $T_{ij;h}^{s}=\sum_{n=1}^{N_{\tau }}\Theta_{ij;n}^{s}\sigma_{n}(x)$ for i,j=1,2. Here, ${\left\{\phi_{n}(x)\right\}}_{n=1}^{N_{u}}$, ${\left\{\psi_{n}(x)\right\}}_{n=1}^{N_{p}}$ and ${\left\{\sigma_{n}(x)\right\}}_{n=1}^{N_{\tau }}$ are suitable piecewise polynomial functions defined on $\Omega_{h}$, referred to as basis functions. A particular choice of approximations that enforces the stability of the finite element discretization was introduced in [50]. In this case, velocity (and stress) basis functions are chosen to be piecewise quadratic, with pressure chosen to be piecewise linear.

The nonlinear problem described in the system (3.10) can be solved iteratively. A Picard iterative process is chosen since it is simple and computationally cheap to implement on a per-iteration basis. This makes it well-suited for the simulations presented in this paper, which we later demonstrate require only a small number of iterations to converge (typically less than 10). The Picard iterative process reads as follows: for each iteration r, we solve


(3.11a)−a(Phs,r,vh)+b(Ths,r,vh)=−d(vh),(3.11b)a(qh,Uhs,r)=0,Δtc0(Ths,r,θh)+αWiΔtc1(Ths,r;Ths,r−1,θh)+Wic2(Ths,r+τNs,r,θh)+WiΔth0(Uhs,r;Ths,r−1,θh)(3.11c)−WiΔth1(Uhs,r;Ths,r−1,θh)−2Δtb(θh,Uhs,r)=Wic2((Ths−1,r+τNs−1,r),θh),

where the initial guess for the stress tensor is given by the equivalent solution to the Newtonian problem. The Picard iterative process may fail to converge for high levels of nonlinearity, e.g. high values of α or Wi. In such cases, we utilize a relaxed Picard iteration in which the solution at the iteration r is updated according to $T_{h}^{s,r}=\nu T_{h}^{s,r}+(1-\nu )T_{h}^{s,r-1}$ for the solution to approach more slowly and avoid ‘overshooting’. Here, 0<ν≤1 is a parameter that determines the speed of approach and is adjusted as required for convergence for each (α,Wi) pair. The problem is deemed converged when $\|T_{h}^{s,r}-T_{h}^{s,r-1}{\|}_{2}<\delta_{\text{tol}}$, with $\|\cdot {\|}_{2}$ representing the Euclidean norm (2-norm) and $\delta_{\text{tol}}$ a tolerance, set in what follows as $\delta_{\text{tol}}=1\times 10^{-3}$.

To simulate the three-sphere swimmer in a fluid defined by parameters (α,Wi), we first choose a domain Ω and generate a mesh of this domain consisting of rectangular elements. We prescribe the initial configuration of the swimmer through choosing ${\left\{X^{\left[m\right]}(0)\right\}}_{m=1}^{3}$ and setting values for the remaining swimmer parameters A, κ, χ, ϵ and ℓ. Given this initial set-up, we calculate the forces ${\left\{f^{\left[m\right]}(0)\right\}}_{m=1}^{3}$, following which the regularized Stokeslet solutions $p_{N}$, $u_{N}$ and $\tau_{N}$ can be calculated at all mesh nodes (via the system (3.6)). Using the Picard iterative process described above, we then calculate the non-Newtonian corrections P, U and T. To construct the system (3.11), we evaluate all integrals using Gauss–Legendre quadrature. The integrals involving the known Newtonian solutions are more computationally costly to evaluate, and we utilize an improved quadrature technique introduced in [51], which reduces the number of quadrature points required by moving the points of near singularity away from the interval of integration through an appropriate mapping. Through applying the basis function expansions and calculating all integrals, we obtain a linear system to solve for the non-Newtonian terms. This can be constructed and solved in MATLAB® using the backslash command ‘∖’ to obtain these solutions at all mesh nodes. To evolve the swimmer, we solve the initial value problem


(3.12) $$\begin{array}{lcr} & \frac{dX^{\left[m\right]}(t)}{dt}=u(X^{\left[m\right]}(t),t),\;X^{\left[m\right]}(0)={X_{0}}^{\left[m\right]}, & \end{array}$$

for m=1,2,3 where ${\left\{{X_{0}}^{\left[m\right]}\right\}}_{m=1}^{3}$ are the prescribed initial values of the three spheres. The velocity at the sphere locations $u\left(X^{\left[m\right]}(t),t\right)=U\left(X^{\left[m\right]}(t),t\right)+u_{N}\left(X^{\left[m\right]}(t),t\right)$ can be found through interpolating using the velocity basis functions ${\left\{\phi_{n}(x)\right\}}_{n=1}^{N_{u}}$ to calculate $U\left(X^{\left[m\right]}(t),t\right)$ and finding $u_{N}\left(X^{\left[m\right]}(t),t\right)$ directly from equation (3.6).

**Figure 2.**
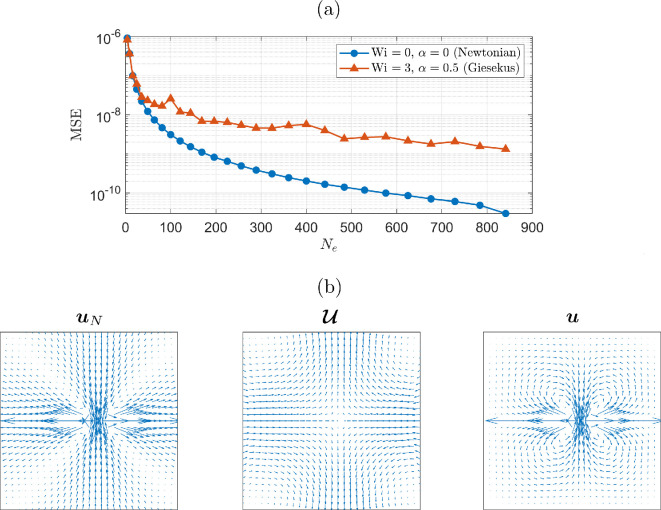
(a) Convergence of the velocity solution with the number of mesh elements $N_{e}$ for a Newtonian and a Giesekus fluid. For a refined mesh with $N_{e}=900$, we plot the mean-squared error over all refined mesh nodes, between the velocity solutions in this case and the interpolated velocity solutions for a less refined mesh with $N_{e}$ elements. (b) The three velocity solutions $u_{N}$, U and u ($=U+u_{N}$) for a fluid with Wi=5 and α=0.5. Results are for a three-sphere swimmer with A=0.8, κ=0.8, χ=π/2, ϵ=0.01 and ℓ=0.2, plotted at t=0.

To investigate the number of elements required in the discretization of the domain Ω for an accurate flow solution, we plot the convergence of the solution with the number of mesh elements $N_{e}$ in figure 2a. Here, we calculate the mean-squared error between the velocity solution over a mesh with $N_{e}$ elements and the solution over a refined mesh with $N_{e}=900$. Figure 2a demonstrates good convergence of the solution, and in all subsequent results presented in this paper, we select $N_{e}=196$. This ensures fast simulations with a good degree of accuracy.

In figure 2b, we plot the velocities $u_{N}$, U and u over Ω for a Giesekus fluid with Wi=5 and α=0.5. The non-Newtonian correction term U remains slowly varying throughout Ω, in contrast with the rapidly varying Newtonian approximation $u_{N}$. The total velocity $u=U+u_{N}$ accounts for the non-Newtonian nature of the fluid and the boundary conditions on ∂Ω. This demonstrates that the hypothesis that the non-Newtonian correction term is slowly varying holds even for quite high values of α and Wi. We now proceed to verify our numerical solver before examining the effects of shear-thinning rheology and viscoelasticity on propulsion.

### (b) Verification

To verify the numerical solver, we compare the output to the solution obtained through semi-analytic techniques for a simple flow problem in the absence of the swimmer. We consider the flow of a Giesekus fluid between two parallel plates separated by a distance h. The bottom plate is stationary, and the top plate moves at a speed V. Under steady, laminar and fully developed flow assumptions, the axial velocity u is the only non-zero velocity component and is a function of y only. The dimensionless problem to solve is given by


(3.13a)−∇p+∇⋅τ=0,∇⋅u=0,(3.13b)τ+αWi(τ⋅τ)−Wi(τ⋅(∇u)+(∇u)T⋅τ)=2D(u),

where u(0)=0 and u(1)=1. Here, the Weissenberg number is defined as Wi=λV/h, obtained through choosing the length scale h, velocity scale V and pressure and stress scales h/ηV. An analytic solution to this problem can be obtained by following Raisi *et al*. [52] as


(3.14) $$\begin{array}{lcr} & u(y)=-\frac{1}{2\alpha G{\text{Wi}}^{2}}\left[\frac{2(\alpha -1)}{1-\alpha {\text{Wi}}^{2}(\tau_{0}+Gy{)}^{2}}+(2\alpha -1)\;\ln\left(1-\alpha {\text{Wi}}^{2}(\tau_{0}+Gy{)}^{2}\right)\right]+C. & \end{array}$$

The constant C can be obtained through applying the first boundary condition above. In equation (3.14), $G=\frac{h^{2}}{\eta V}\left(\frac{dp}{dx}\right)$ is the dimensionless pressure drop and $\tau_{0}$ is the dimensionless shear stress at the fixed plate, found through solving


(3.15) $$\begin{array}{lcr} & \frac{2\alpha -1}{2\alpha {\text{Wi}}^{2}G}\ln\left(\frac{1-\alpha {\text{Wi}}^{2}\tau_{0}^{2}}{1-\alpha {\text{Wi}}^{2}{\left(\tau_{0}+G\right)}^{2}}\right)-\frac{\left(\alpha -1)(G+2\tau_{0}\right)}{\left(1-\alpha {\text{Wi}}^{2}\tau_{0}^{2})(1-\alpha {\text{Wi}}^{2}(\tau_{0}+G{)}^{2}\right)}-1=0, & \end{array}$$

which is derived through applying the boundary conditions to equation (3.14). Equation (3.15) is strongly nonlinear but can be solved using the Newton–Raphson method. Raisi *et al*. calculated the velocity solutions over α∈[0.1,0.5] and Wi∈[1,5] for G=1, with the Newtonian value of $\tau_{0}$ used as an initial guess for the numerical scheme (${\tau_{0}}_{\text{init}}^{1}=1-G/2$). Our analysis indicates that such an initial guess does not always lead to the profiles reported. In particular, our semi-analytic results diverge from those of Raisi *et al*. for Wi≥3. Furthermore, the form of the velocity profile can depend heavily on the initial guess for $\tau_{0}$, demonstrating the existence of multiple solutions to the flow problem. We choose to investigate these multiple solutions further before verifying our numerical implementation of the Giesekus solver to understand the flow problem in detail.

**Figure 3.**
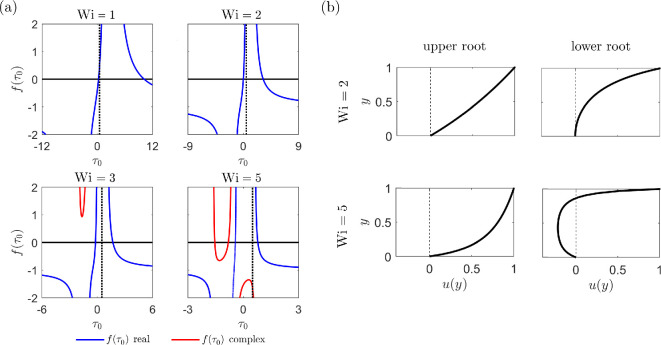
(a) Value of the function $f(\tau_{0})$ with the dimensionless shear stress $\tau_{0}$, indicating the presence of multiple solutions to the nonlinear equation (3.15). Results are shown for α=0.1, G=1 and a range of Weissenberg numbers Wi={1,2,3,5}. Regions in which the value of $f(\tau_{0})$ is real are shown in blue, with complex regions indicated in red. Dotted lines indicate the value of the initial guess for $\tau_{0}$ used by Raisi *et al.* [52] in the Newton–Raphson solver. (b) Velocity profiles u(y) corresponding to both roots from (a) and two choices of Weissenberg number, Wi={2,5}.

We analyse the roots of the function $f\;(\tau_{0})$ defined as the left-hand side of equation (3.15). The value of $f\;(\tau_{0})$ over a range of $\tau_{0}$ is plotted in figure 3a for the choices of Wi, α and G considered in Raisi *et al*. Regions in which the value of $f(\tau_{0})$ is real are plotted in blue, with complex regions indicated in red. We observe that precisely two real roots of the function exist over all values of Wi considered. The initial value ${\tau_{0}}_{\text{init}}^{1}=1-G/2$ is indicated in figure 3a via a dotted line. For lower values of the Weissenberg number (Wi=1 and Wi=2), this choice of initial guess for $\tau_{0}$ leads to the nonlinear solver converging on the lower of the two roots of the function $f(\tau_{0})$. For Wi=3 and Wi=5, the solver converges on the higher of the roots. The results in Raisi *et al*. depict the velocities resulting from choosing the lower root as $\tau_{0}$.

Selecting the lower root in figure 3a is essential to obtaining a physically relevant velocity profile. To demonstrate this, we plot the velocity profiles for both roots at Wi=2 and Wi=5 in figure 3b. Given G=1>0, pressure increases in the direction of the upper-plate motion, and we expect the forces due to pressure to be acting in the opposite direction. This adverse pressure gradient should lead to a situation where the velocity over a portion of the channel can become negative and back flow may occur near the stationary plane. However, the velocity profiles for the upper root case show a situation in which a favourable pressure gradient is present, which is incorrect based on the choice of G. For the lower root, plotting the same velocity profiles leads to the desired results for the adverse pressure gradient case. However, this root only takes a real value up to approximately Wi≈6, past which it is complex. In this way, a real and physically relevant solution to this problem may only be obtained up to Wi≈6 (for the case where α=0.1 and G=1).

Our analysis of this semi-analytic solution has revealed the existence of multiple solutions to equation (3.15), which were previously not discussed by Raisi *et al*. [52]. We have uncovered that to obtain the desired physically relevant velocity profiles, we must use the lower root of equation (3.15) depicted in figure 3a. Our initial guess for the dimensionless shear rate at the stationary plate $\tau_{0}$ must therefore be chosen for the nonlinear solver to converge on this lower root. This will then be used to verify our numerical (FEM) solution of the Giesekus problem.

To solve the same problem using the FEM, we consider a slight modification of the derivation outlined in §3a, where the domain boundary ∂Ω can be decomposed into two disjoint parts $\partial \Omega_{N}$ and $\partial \Omega_{D}$ where Neumann and Dirichlet boundary conditions are applied, respectively. We decompose the boundary $\partial \Omega_{D}$ into three components via $\partial \Omega_{D}=\Gamma_{1}\cup \Gamma_{2}\cup \Gamma_{3}$, where $\Gamma_{1}$, $\Gamma_{2}$ and $\Gamma_{3}$ represent the top wall, bottom wall and channel inlet, respectively. The dimensionless Dirichlet boundary conditions on these walls are given by $u=[1,0{]}^{T}$ on $\Gamma_{1}$, u=0 on $\Gamma_{2}$ and $u=u_{\text{in}}$ on $\Gamma_{3}$. The boundary condition applied at the inlet specifies that the velocity on $\Gamma_{3}$ is equal to that calculated via the semi-analytic solution given in equation (3.14), labelled $u_{\text{in}}$. At the outlet $\partial \Omega_{N}$, the zero normal stress condition τ⋅n=0 is applied where n is the outwards-pointing unit normal to the boundary.

**Figure 4.**
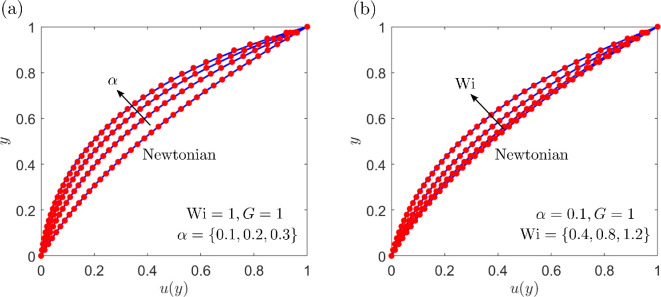
Profiles of the dimensionless velocity u with y over changing (a) α={0.1,0.2,0.3}, where Wi=1, and (b) Wi={0.4,0.8,1.2}, where α=0.1. In both cases G=1, and the Newtonian solution is also plotted. Analytic solutions are shown in blue with numerical (FEM) solutions shown with red markers.

The Picard iterative process is applied to solve the problem, where we note that in the absence of a swimmer, $p_{N}$, $u_{N}$ and $\tau_{N}$ are all equal to zero. We choose a Newtonian initial guess for the stress tensor, which allows the solver to converge on the physically correct (lower) root, demonstrated in figure 3, for the values of Wi considered (Wi∈{0.4,0.8,1.2}). A comparison between the numerical (FEM) and semi-analytic flow profiles is shown in figure 4. In figure 4a, we plot the change in the velocity over α for Wi=1 and G=1. Similarly, the profiles over Wi are shown in figure 4b, where α=0.1. Increasing α (shear-thinning effects) or Wi (fluid elasticity) leads to increased velocity gradients. All profiles show good agreement between the numerical and semi-analytic solutions. Here we only consider the ranges α∈[0,0.3] and Wi∈[0,1.2], since for this particular flow problem, the Picard iterative process struggles to converge past this point. However, in our later results, we are able to extend this range.

## 4. Simulation of the three-sphere swimmer in a Giesekus fluid

We now explore the effect of a Giesekus fluid on the propulsion of the three-sphere swimmer. This is quantified through examining the swimming speed and efficiency of the swimmer for different values of α and Wi. The swimming speed of the swimmer can be defined as the net translation of any reference point on the swimmer (in our case, the position of the central sphere $X^{\left[2\right]}$) per one beat cycle. In this way, we define


(4.1) U(j)=‖X[2](j)−X[2](j−1)‖/T,

where T is the period and $X^{\left[2](j\right)}$ represents the position of the central sphere after j periods. The Lighthill efficiency of the swimmer [53] is calculated via


(4.2) E(j)=(U(j))2/P(j) with P(j)=1T∫(j−1)T(j)TU⋅Fdt,

where P(j) is the total power expended by the swimmer over the jth period with $U=[u(X^{\left[1\right]}(t)),u(X^{\left[2\right]}(t)),u(X^{\left[3\right]}(t)){]}^{T}$ and $F=[f^{\;[1]}(t),f^{\;[2]}(t),f^{\;[3]}(t){]}^{T}$.

In what follows, we simulate a three-sphere swimmer defined by parameters A=0.8, κ=0.8, χ=π/2, ϵ=0.01 and ℓ=0.2. These values are chosen to produce a progressive beat with quick convergence of the iterative process over all values of α and Wi considered ( less than 10 iterations). The initial coordinates of the spheres are prescribed as $X^{\left[1\right]}(t=0)=[0.8,1{]}^{T}$*,* $X^{\left[2\right]}(t=0)=[1,1{]}^{T}$ and $X^{[3}(t=0)=[1.2,1{]}^{T}$, and the swimmer is situated within a domain Ω=[0,2]×[0,2]. We consider values α∈[0,0.5] for the shear-thinning index and Wi∈[0,5] for the Weissenberg number. Past Wi=5, the numerical method may struggle to converge. This problem could be alleviated through formulating the constitutive model in terms of the logarithm of the conformation tensor [54], which improves numerical convergence for high Weissenberg numbers. This is, however, beyond the scope of this paper and presents an opportunity for future work. Simulations are run over 125 beats before the propulsive measures (U(125) and $E^{\left(125\right)}$) are calculated so that the swimmer has developed a stable beat.

**Figure 5.**
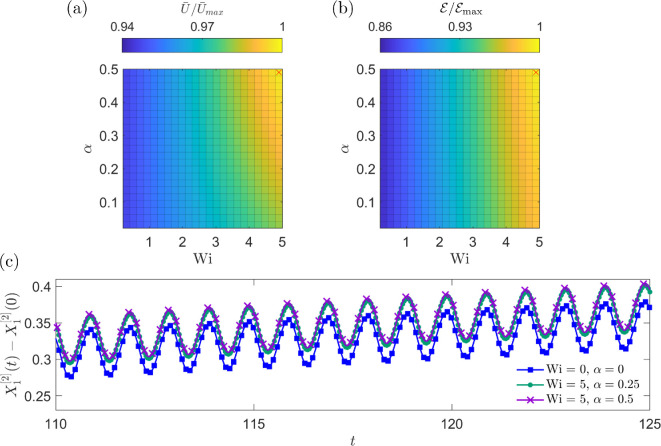
The effect of Wi∈[0,5] and α∈[0,0.5] on (a) normalized swimming speed (U) and (b) normalized Lighthill efficiency (E) of the three-sphere swimmer. Values are normalized with respect to the maximum (a) swimming speed and (b) efficiency, which occur at the locations marked by a red x. (c) Comparison of the trajectory of the *x*-coordinate of the central sphere of the swimmer over the final 15 beats of the simulation for several different values of Wi and α. The Newtonian case is indicated in blue, where Wi=0 and α=0. Positions are given relative to the initial location of the central sphere at t=0, given by $X_{1}^{\left[2\right]}(0)=1$.

The effects of varying the shear-thinning parameter α and the Weissenberg number Wi on swimming speed and efficiency are shown in figure 5a,b. The maximum $\bar{U}$ and E are obtained when Wi=5 and α=0.5, corresponding to a fluid with the strongest shear-thinning and viscoelastic effects over the parameter ranges considered. Conversely, the swimmer with the lowest measured $\bar{U}$ and E occurs in the Newtonian case where Wi=α=0. Swimming speed and efficiency both increase monotonically with Wi across all values of α, indicating that fluid elasticity aids propulsion of the swimmer. Speed and efficiency increases of up to approximately 7 and 16%, respectively, are observed, compared to swimming in a Newtonian fluid. The shear-thinning parameter α also has a clear, although less significant, effect on propulsion. For low values of Wi, α has little effect on $\bar{U}$ and E; however, as Wi increases, the influence of α becomes more pronounced. Increasing α also leads to monotonic increases in swimming speed and efficiency of the swimmer (although very small for efficiency), indicating that shear-thinning rheology also enhances propulsion, with this effect magnified for fluids with higher levels of elasticity.

The influence of Wi and α on the trajectory of the swimmer is explored in figure 5c. The shape of the paths traced by the swimmer over each beat for the Newtonian case (Wi=α=0) and the Giesekus cases (Wi=5 with α=0.25 or α=0.5) is almost identical. The progress of the swimmer is, however, significantly higher for the Giesekus cases compared with the Newtonian case, further demonstrating the enhancement in propulsion of the swimmer with increased non-Newtonian effects. Between the two cases where Wi=5, progress is slightly higher when α=0.5 compared with α=0.25, showing the more subtle effect of shear-thinning rheology to enhance propulsion. In these simulations, $N_{b}=125$ beats have been calculated, and it is expected that the differences in trajectory would become more pronounced for higher values of $t_{\text{max}}$.

**Figure 6.**
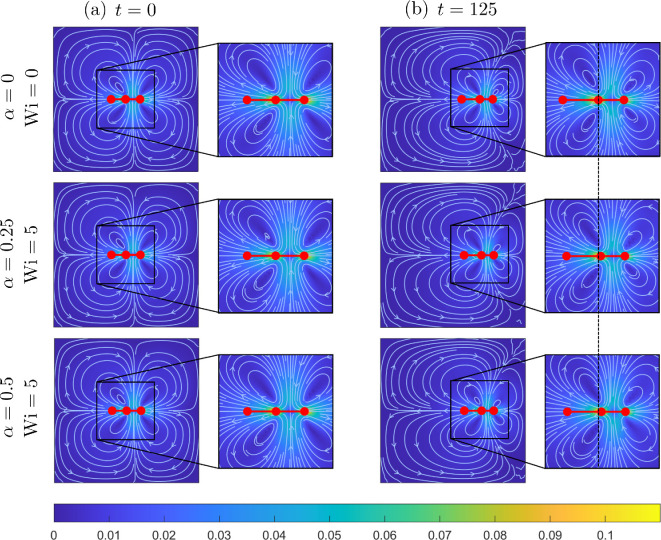
Comparison of the flow fields surrounding the three-sphere swimmer in a Newtonian fluid ((α,Wi)=(0,0)) and two Giesekus fluids with (α,Wi)=(0.25,5) and (α,Wi)=(0.5,5). Results are plotted at two points at (a) t=0 and (b) t=125. Fluid velocity magnitude is indicated by the colourbar, and instantaneous fluid streamlines are shown in white with arrows indicating direction.

To analyse the effect of shear-thinning rheology and viscoelasticity on propulsion, we examine the velocity fields, stress distribution and viscosity surrounding the swimmer for different values of α and Wi. In figure 6, we plot the velocity fields associated with the swimmer at the beginning (t=0) and end (t=125) of the simulation for the pairs of (α,Wi) considered in figure 5c. Here, we observe very little variation in the fluid streamlines or velocity magnitude surrounding the swimmer for the Newtonian case versus the non-Newtonian cases, suggesting that shear-thinning rheology and fluid elasticity have little effect on the overall flow direction or strength.

**Figure 7.**
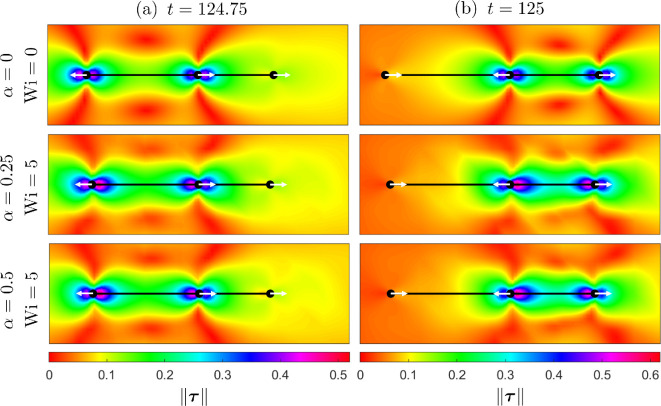
Comparison of stress distribution ‖τ‖ surrounding the three-sphere swimmer in a Newtonian fluid ((α,Wi)=(0,0)) and two Giesekus fluids with (α,Wi)=(0.25,5) and (α,Wi)=(0.5,5). Results are plotted at two points over the final beat cycle (a) t=124.75 and (b) t=125. White arrows show the direction of travel of the three spheres at these time points.

To uncover the influence of fluid elasticity on propulsion, we examine the polymer stress induced by the motion of the swimmer. In figure 7, we plot polymer stress energy, computed as the magnitude of the polymer stress tensor, across three values of the parameter pair (α,Wi) and at two times over the final beat t∈[124,125]. Polymer stress builds up quickly, typically within 5–10 full beat cycles of the swimmer. In this way, plotting the polymer stress over the final beat ensures the stress is fully developed. Across both time points, we observe a higher concentration of stresses in the region behind each sphere (compared to the direction of motion, indicated by white arrows) in the high fluid elasticity cases compared to the Newtonian case. At t=124.75, we observe approximately a 9–10% increase in the maximum stress across the domain in a Giesekus fluid compared to the Newtonian case. For t=125, we see approximately a 17–19% increase. Overall, this demonstrates that across each beat cycle, there is a significant buildup of stressed fluid in non-Newtonian fluids versus their Newtonian counterpart. We hypothesize that this stressed region could lead to the increases in propulsion observed in figure 5 with fluid elasticity, similar to previously observed results for undulatory swimmers in purely viscoelastic fluids [17].

**Figure 8.**
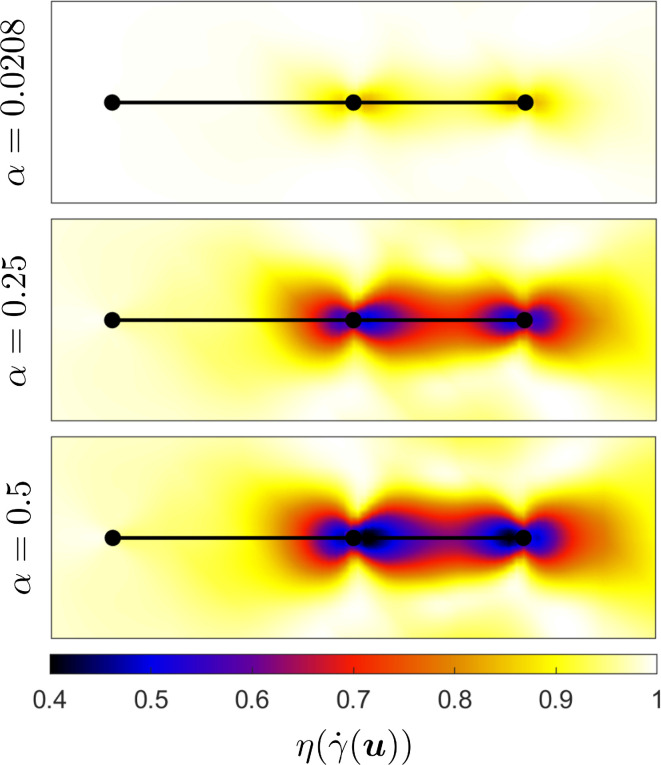
Comparison of the dimensionless viscosity distribution $\eta (\dot{\gamma }(u))$ surrounding the three-sphere swimmer in a Giesekus fluid with Wi=5 and three different values of α∈{0.0208,0.25,0.5}. Results are plotted at t=125.


While in a Newtonian fluid, the swimmer experiences an environment of constant viscosity, we expect to see regions of thinned fluid surrounding the swimmer in a Giesekus fluid. In figure 8, we plot the viscosity surrounding the cell for Wi=5 and three different choices of α∈{0.0208,0.25,0.5}. The viscosity for a Giesekus fluid is calculated via the material function $\eta =\left(1-f{)}^{2}/(1+(1-2\alpha )f\right)$ [47], where

(4.3) $$\begin{array}{lcr} & f=\frac{1-\chi }{1+(1-2\alpha )\chi },\;\chi^{2}=\frac{(1+16\alpha (1-\alpha )(\text{Wi}\;\dot{\gamma }{)}^{2}{)}^{1/2}-1}{8\alpha (1-\alpha (\text{Wi}\;\dot{\gamma }){)}^{2}}. & \end{array}$$

Over each value of α, we observe a region of thinned fluid surrounding the swimmer. As we increase α, the fluid becomes more shear-thinning, and we observe an increase in the size of the shear-thinning region and a reduction in viscosity close to the swimmer. The thinned envelope surrounding the swimmer is reminiscent of that observed in other studies of swimmers in shear-thinning fluids [13,37,55]. The slight increase in swimming speed with α observed in figure 5 may be due to a confinement-like effect, where swimmers propel slightly faster when contained in a region of low viscosity fluid surrounded by a region of high viscosity fluid. This mechanism has been proposed previously for swimming enhancement in shear-thinning fluids [21,22,35,56].

## 5. Discussion

Many microswimmers navigate non-Newtonian environments, but only fairly recently have we begun to investigate how these complex fluids affect their propulsion. Our understanding remains limited in part due to the lack of flexible and computationally efficient modelling approaches for the nonlinear and rapidly varying flow problems inherent to non-Newtonian rheology. Such approaches would allow us to explore the transitions between the apparently contradictory results in the literature.

In this paper, we introduced the HANS method for modelling a simple conceptual three-sphere swimmer in a Giesekus fluid, which exhibits shear-thinning and viscoelastic effects. HANS utilizes the MRS as an approximation to the flow around the swimmer, which is then ‘corrected’ through solving for a non-Newtonian term via an iterative finite element approach. Because the flow correction is slowly varying across the computational domain (demonstrated in figure 2b), we can achieve accurate results over a coarse mesh (figure 2a) without requiring a body-fitted mesh like previous methods [13]. This approach reduces computational expenses compared to directly solving the full nonlinear flow problem, enabling efficient simulations of microswimmers in non-Newtonian environments. In the absence of a swimmer, we verified our implementation of the FEM solver for a Giesekus fluid by comparing our solutions with those found through a semi-analytic approach [52] for Couette–Poiseuille flow.

Modelling using the Giesekus constitutive law enabled us to investigate how shear-thinning and fluid elasticity influence the propulsion of a swimmer with fixed beat parameters (A=0.8, κ=0.8, χ=π/2). We found that both properties enhance swimming speed and efficiency by up to 16% over 125 beats, with viscoelasticity having the most significant effect. This is qualitatively consistent with several experimental studies that report enhanced swimming in non-Newtonian fluids [18–20,22,23,57,58]. For example, human sperm have been shown to exhibit greater displacement per beat when swimming in more viscoelastic environments [57]. Similarly, bovine sperm demonstrate significant improvements in thrust efficiency when moving through shear-thinning viscoelastic fluids compared to Newtonian fluids [58]. These effects are more pronounced in these studies than in our own results, probably due to differences in swimmer morphology.

While the shear-thinning parameter α and Weissenberg number Wi do not significantly alter the flow field surrounding the swimmer, we observed that they do affect stress and viscosity distributions, affecting propulsion. Other theoretical studies have similarly reported speed enhancements in fluids exhibiting either shear-thinning or viscoelastic effects [13–17,21]. Therefore, it is perhaps not surprising that incorporating both properties leads to additional boosts in swimmer performance. Moreover, recent numerical studies on collective motion in a Giesekus fluid [59] indicate that these effects can significantly influence swimmer behaviour, including aiding cluster formation and enhancing swimming speed for spherical puller swimmers. In future work, it would be interesting to explore whether these conclusions are independent of the particular nonlinear constitutive law chosen for the fluid. The Phan–Thien–Tanner model [60], for example, exhibits similar properties to the Giesekus model, although it differs in its prediction of zero second normal stress difference for shear flow in non-circular channels.

One of the current limitations of the HANS method is that it exhibits the ‘high Weissenberg number problem’, that is, issues with convergence past the values of Wi considered in this paper. In future work, we wish to address this by formulating the problem via the logarithm of the conformation tensor, which has been demonstrated to significantly improve convergence [54]. Other future studies will employ the HANS method to model various microswimmers, such as bacteria, sperm cells and algae, and investigate the effects of complex fluids on propulsion in these cases. In such work, it would be interesting to explore the interplay between the beat dynamics and propulsive gains of swimmers in non-Newtonian fluids. This could, for instance, reveal whether there is an optimal swimming stroke for a fluid exhibiting specific shear-thinning effects or fluid elasticity and provide insight into which cells are best adapted to their biological environment.

Designed for flexibility, the HANS method allows for the adaptation to different swimmer types by simply modifying the Newtonian flow approximation, rather than necessitating a complete reformulation of the problem. This flexibility represents a significant advantage of this modelling approach, which can also easily be extended to three dimensions. In terms of computational complexity, modelling more complex bio-inspired swimmers or multiple swimmers should not require significant changes to the finite element mesh and, therefore, should not alter the size of the linear system being solved. However, it is of note that the problem will become significantly more computationally expensive to solve as the number of regularized Stokeslets (resolution/number of swimmers) increases, which would probably eventually limit what problems can be solved using the given hardware. Finally, future work will also use HANS to model swimmers in complex domains or near boundaries. The HANS method is not currently accurate for swimmers near boundaries due to the choice of image system (the Stokeslet). Through employing a modified image system for a point force near a plane wall [61], HANS could, for example, be used to explore the behaviour of bacteria near surfaces in non-Newtonian fluids, aiding in understanding the influence of complex fluids on the early stages of biofilm formation.

## Data Availability

Code to calculate the results in this paper can be accessed at [62].
